# Comparison of long-term outcomes between enteral nutrition via gastrostomy and total parenteral nutrition in older persons with dysphagia: A propensity-matched cohort study

**DOI:** 10.1371/journal.pone.0217120

**Published:** 2019-10-02

**Authors:** Shigenori Masaki, Takashi Kawamoto

**Affiliations:** 1 Shigenori Masaki, Department of Surgery and Gastroenterology, Miyanomori Memorial Hospital, Sapporo, Hokkaido, Japan; 2 Takashi Kawamoto, Department of Neurosurgery, Miyanomori Memorial Hospital, Sapporo, Hokkaido, Japan; University of Malaya Faculty of Medicine, MALAYSIA

## Abstract

**Background:**

The long-term outcomes of artificial nutrition in older people with dysphagia remain uncertain. Enteral nutrition via percutaneous endoscopic gastrostomy (PEG) is one of the major methods of artificial nutrition. Enteral feeding is indicated for patients with a functional gastrointestinal tract. However, total parenteral nutrition (TPN) is often inappropriately chosen for artificial nutrition in Japan, even in patients with a functional gastrointestinal tract, as PEG has recently been viewed as an unnecessary life-prolonging treatment in Japan. This study aimed to compare the long-term outcomes between PEG and TPN.

**Methods:**

This single-center retrospective cohort study investigated long-term outcomes in older patients with dysphagia who received PEG or TPN between January 2014 and January 2017. The primary outcome was survival time. Secondary outcomes were oral intake recovery, discharge to home, and the incidence of severe pneumonia and sepsis. We performed 1-to-1 propensity score matching using a 0.05 caliper. The Kaplan–Meier method, log-rank test, and Cox regression analysis were used to compare the survival time between groups.

**Results:**

We identified 253 patients who received PEG (*n* = 180) or TPN (*n* = 73). Older patients, those with lower nutritional states, and severe dementia were more likely to receive TPN. Propensity score matching created 55 pairs. Survival time was significantly longer in the PEG group (median, 317 vs 195 days; *P* = 0.017). The hazard ratio for PEG relative to TPN was 0.60 (95% confidence interval: 0.39–0.92; *P* = 0.019). There were no significant differences between the groups in oral intake recovery and discharge to home. The incidence of severe pneumonia was significantly higher in the PEG group (50.9% vs 25.5%, *P* = 0.010), whereas sepsis was significantly higher in the TPN group (10.9% vs 30.9%, *P* = 0.018).

**Conclusions:**

PEG was associated with a significantly longer survival time, a higher incidence of severe pneumonia, and a lower incidence of sepsis compared with TPN.

## Introduction

Artificial nutrition is a medical intervention for patients suffering from dysphagia due to various clinical conditions. Artificial nutrition is administered via the enteral if the gastrointestinal tract is functioning. Percutaneous endoscopic gastrostomy (PEG) feeding is one of the major methods of artificial nutrition. PEG was initially developed as an enteral feeding technique for pediatric patients with dysphagia [1,2]. Compared to feeding via a nasogastric tube, enteral feeding via PEG can relieve laryngopharyngeal discomfort and prevent intervention failure; therefore, its use has become widespread for long-term enteral feeding in multiple patient groups including pediatric and geriatric populations [3,4]. In the past, studies have reported worse outcomes following PEG feeding in patients with dementia [5,6]. A recent study showed that the survival duration is greater among dementia patients with PEG feeding than among those without tube feeding [7].

TPN is another common method of nutritional management [8]. TPN is usually indicated if the gastrointestinal tract is non-functional [9]. TPN is not an alternative to PEG feeding in individuals with a functional gastrointestinal tract. However, TPN is occasionally used in Japan for artificial nutrition in older patients with dysphagia regardless of a functional gastrointestinal tract [10]. TPN is more expensive compared to enteral nutrition [11]. Therefore, the cost of long-term artificial nutrition using TPN can be a financial burden for patients and their family members. However, in Japan, 90% of medical expenses in people aged 75 years or older is covered by a medical insurance system for older senior citizens [12]. Hence, the cost of TPN does not tend to become a barrier in the choice of TPN for long-term artificial nutrition. On the other hand, national medical expenses in Japan have been increasing with the expansion of the aging population. Japan is currently struggling to obtain the necessary funds and to reduce medical expenses [12].

The Japan Geriatrics Society published a position statement on end-of-life care for older patients in 2012, suggesting that withholding or withdrawing from feeding tubes are viable options [13]. The Ministry of Health, Labour and Welfare in Japan lowered medical treatment fees for PEG in 2014 [14]. As a result, the general population in Japan has come to view PEG as being representative of unnecessary life-prolonging treatment [15], despite the fact that both PEG and TPN can be life-prolonging treatments [10]. PEG is generally avoided in older patients; hence, a greater number of older patients with dysphagia choose TPN instead of PEG feeding for long-term artificial nutrition [15]. The long-term outcomes of PEG feeding versus TPN in older patients with dysphagia have previously been poorly documented. Therefore, we aimed to compare the long-term outcomes of PEG feeding and TPN in older patients using propensity score-matched analysis [16].

## Methods

### Study design

This study was a single-center, retrospective cohort study using propensity score-matched analysis. Consecutive older patients with dysphagia who underwent PEG using the modified introducer method [17] or TPN including implantable central venous ports (PORT), non-tunneled central venous catheters (NT-CVC), and peripherally inserted central catheters (PICC) for long-term artificial nutrition between January 2014 and January 2017 were considered for inclusion in the study. We excluded patients who had advanced cancer and those who required a PEG for gastric decompression. We also excluded TPN patients who had a PEG inserted before January 2014. Patients who received both PEG feeding and TPN between January 2014 and January 2017 were assigned to the PEG group.

The decision to move forward with PEG feeding or TPN was made after sufficient discussion, including withholding artificial nutrition between patients or their family and clinicians. We investigated the percentage of the patients who exerted their autonomy and took part in the decision-making process of choosing between PEG feeding and TPN and the percentage of the patients whose family members made the final decision. With regards to TPN cases, the choice between PORT, NT-CVC, and PICC was made based on the patient’s or their family’s request and the feasibility and acceptability of each catheter in the discharge destination. Appropriate nutrition was administered based on clinical evaluation by clinicians. Clinical details were obtained from patients’ medical charts including age, gender, height, weight, underlying diseases, and blood test results. We used blood test results performed within 7 days before the start of PEG feeding or TPN. Body mass index (BMI) was calculated using the height and weight measured on admission. We investigated daily calorie intake on the seventh day after the procedure in both groups. We calculated the median (interquartile range; IQR) values for BMI and daily calorie intake. We investigated the Clinical Frailty Scale (CFS) at the point of insertion of the PEG tubes or TPN catheters [18].

Because of the anonymous nature of the data, the requirement for informed consent was waived. Study approval was obtained from the Ethical Review Board of Miyanomori Memorial Hospital.

### Outcomes

The primary outcome was defined as survival time after the start of the procedure. The secondary outcomes included oral intake recovery, discharge to home, and the incidence of severe pneumonia and sepsis. Oral intake recovery was defined as withdrawal from PEG feeding or TPN for at least 1 month during the observational period. Discharge to home included discharge to private residential homes and housing with health and welfare services for older persons. Definitions of oral intake recovery and discharge to home were based on that of the Ministry of Health, Labour and Welfare of Japan [19]. The diagnosis of severe pneumonia and sepsis was based on clinical diagnosis by trained physicians. The most frequent cause of death in both the PEG and TPN groups was investigated.

### Statistical analysis

We used propensity score matching to adjust baseline differences between the groups [16]. The propensity score was calculated by logistic regression for estimating the probability that a patient would receive PEG feeding or TPN. We defined the following variables as potential confounders: age, gender, underlying diseases (cerebrovascular diseases, severe dementia, neuromuscular diseases, previous history of aspiration pneumonia, ischemic heart diseases, chronic heart failure, chronic lung diseases, chronic liver diseases, and chronic kidney diseases), and laboratory values (serum albumin, total lymphocyte count [TLC], total cholesterol [TC], hemoglobin, and C-reactive protein) [20–23]. We performed multiple imputation to handle missing data. We created and analyzed 20 multiply imputed data sets [24,25]. The area under the receiver operating characteristic (ROC) curve was created to evaluate the performance of the logistic regression model for estimating the propensity score [26]. One-to-one propensity score matching was performed to compare the primary and secondary outcomes between the 2 groups using a 0.05 caliper, equal to 0.2 of the standard deviation of the logit of the propensity score [27,28].

We examined patient characteristics before and after propensity score matching between the groups. Continuous variables were compared with the use of the t-test or the Mann–Whitney U test, as appropriate, and categorical variables were compared with the use of Fisher’s exact test between the groups.

Survival was estimated with the Kaplan–Meier method, and the survival rate was compared using the log-rank test. We performed subgroup analysis for survival to investigate the effect of age, gender, cerebrovascular disease, severe dementia, and serum albumin. Data were censored on 28th February 2018. Cox proportional hazards models were used to estimate the hazard ratio (HR) of death for PEG feeding compared to TPN. Logistic regression analyses were used to estimate the odds ratio (OR) of outcomes. The threshold for significance was *P*<0.05. All statistical analyses were conducted using EZR version 1.37, a graphical user interface for R (The R Foundation for Statistical Computing, version 3.4.1) [29]. The Packages 'rms version 5.1–2' and 'Matching version 4.9–3' of the R software were used for multiple imputation and propensity score matching.

## Results

A total of 253 patients met the inclusion criteria, 180 of whom underwent PEG feeding and 73 of whom underwent TPN. From the included patients, 2.8% of patients exerted their autonomy and took part in the decision-making process of choosing between PEG feeding and TPN. In 98.0% of patients, their family members made the final decision regarding PEG feeding or TPN. The TPN group included 28 cases of PORT, 26 cases of NT-CVC, and 19 cases of PICC. The median length of follow-up for censored cases was 601 days (range, 404–823 days).

In the PEG group, missing values for TC were observed in 11 cases (6.1%). In the TPN group, missing TC and TLC values were observed in 1 case (1.4%) and 5 cases (6.8%), respectively. Missing data occurred at random because TC and TLC were not included in routine blood tests in our hospital.

Propensity score matching created 55 pairs in the PEG and TPN groups. The good fit was confirmed by the ROC curve with an area under the curve value of 0.82 (95% confidence interval [CI]: 0.76–0.87). The TPN group included 23 cases of PORT, 18 cases of NT-CVC, and 14 cases of PICC. The baseline characteristics before propensity score matching between the groups are shown in **Table 1**.

**Table 1:** Baseline characteristics of patients before propensity score matching.

Variable	PEG group	TPN group	*P*-value
	(*n* = 180)	(*n* = 73)	
Age (yr)	83	88	<0.001
	(78–88)	(83–90)	
Sex (male)	71 (39.4%)	28 (38.4%)	1.00
Cerebrovascular diseases	107 (59.4%)	26 (35.6%)	0.001
Severe dementia	57 (31.7%)	45 (61.6%)	<0.001
Neuromuscular diseases	10 (5.6%)	4 (5.5%)	1.00
Aspiration pneumonia	73 (40.6%)	21 (28.8%)	0.086
Ischemic heart diseases	31 (17.2%)	16 (21.9%)	0.38
Chronic heart failure	70 (38.9%)	37 (50.7%)	0.093
Chronic lung diseases	12 (6.7%)	7 (9.6%)	0.44
Chronic liver diseases	9 (5.0%)	6 (8.2%)	0.38
Chronic kidney diseases	29 (16.1%)	24 (32.9%)	0.006
Serum albumin (g/dl)	3.3	2.9	<0.001
	(2.9–3.7)	(2.4–3.2)	
Total lymphocyte count (mm^3^)	1236	1058	0.015
	(940–1628)	(699–1505)	
Total cholesterol (mg/dl)	160	142	0.006
	(133–187)	(115–172)	
Hemoglobin (g/dl)	11.3	10.0	<0.001
	(10.2–12.7)	(8.9–11.7)	
C-reactive protein (mg/dl)	0.7	2.0	<0.001
	(0.2–2.9)	(0.7–4.3)	

Values of age, serum albumin, total lymphocyte count, total cholesterol, hemoglobin, and C-reactive protein are median (IQR). Values of other variables are given in numbers (%).

Patients with greater age, severe dementia, chronic kidney disease, lower serum albumin, TLC, TC, and hemoglobin levels, as well as higher C-reactive protein levels were more likely to receive TPN. Patients with cerebrovascular disease were more likely to receive PEG. The baseline characteristics after propensity-score matching between the groups are shown in **Table 2**.

**Table 2:** Baseline characteristics of patients after propensity score matching.

Variable	PEG group	TPN group	*P*-value
	(*n* = 55)	(*n* = 55)	
Age (yr)	86	86	0.76
	(83–90)	(81–90)	
Sex (male)	21 (38.2%)	23 (41.8%)	0.70
Cerebrovascular diseases	18 (32.7%)	20 (36.4%)	0.69
Severe dementia	31 (56.4%)	34 (61.8%)	0.56
Neuromuscular diseases	2 (3.6%)	2 (3.6%)	1.00
Aspiration pneumonia	23 (41.8%)	19 (34.5%)	0.43
Ischemic heart diseases	11 (20.0%)	12 (21.8%)	0.82
Chronic heart failure	30 (54.5%)	25 (45.5%)	0.34
Chronic lung diseases	6 (10.9%)	4 (7.3%)	0.51
Chronic liver diseases	3 (5.5%)	2 (3.6%)	0.65
Chronic kidney diseases	17 (30.9%)	14 (25.5%)	0.53
Serum albumin (g/dl)	2.9	2.9	0.70
	(2.4–3.3)	(2.6–3.2)	
Total lymphocyte count (mm^3^)	999	1111	0.63
	(795–1277)	(708–1481)	
Total cholesterol (mg/dl)	142	143	0.38
	(113–156)	(115–173)	
Hemoglobin (g/dl)	10.3	10.2	0.49
	(8.7–11.1)	(8.9–11.8)	
C-reactive protein (mg/dl)	2.4	2.0	0.76
	(0.3–5.7)	(0.6–5.0)	

Values of age, serum albumin, total lymphocyte count, total cholesterol, hemoglobin, and C-reactive protein are median (IQR). Values of other variables are given in numbers (%).

After propensity score matching, the baseline characteristics were well balanced between the groups.

The median BMI values (IQR) in the PEG and TPN groups, after propensity score matching, were 19.0 (3.3) vs. 18.8 (4.8), respectively. The median daily calorie intake (IQR) was 900 (0) vs. 770 (250) kcal/d, respectively. The number of cases classed as CFS 7 (severely frail) was 7 cases (12.7%) and 6 cases (10.9%), in the PEG and TPN groups, respectively. The number of cases classed as CFS 8 (very severely frail) was 48 cases (87.3%) and 49 cases (89.1%), respectively.

**Fig 1:**
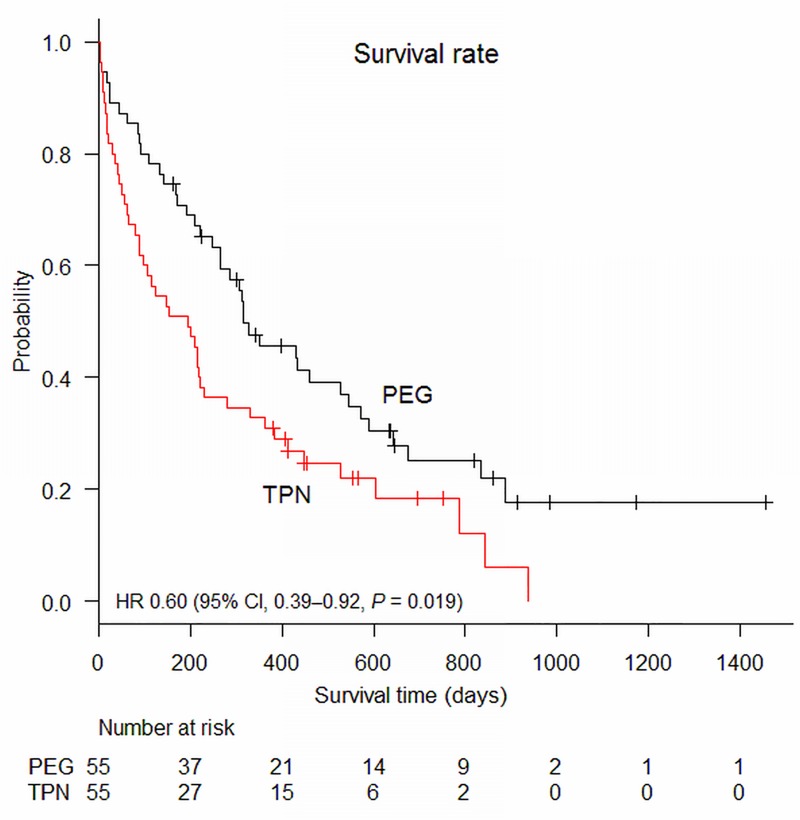
Kaplan–Meier curves of the propensity-matched groups for PEG and TPN. Survival curves of the PEG and TPN groups are shown. Survival time was significantly longer in the PEG group than in the TPN group.

The Kaplan–Meier curve is illustrated in **Fig 1**. The log-rank test showed a significantly longer survival time in the PEG group compared with the TPN group (median, 317 vs 195 days, *P* = 0.017). Cox regression analysis showed that HR for the PEG group relative to the TPN group was 0.60 (95% CI: 0.39–0.92; *P* = 0.019).

The secondary outcomes of propensity-matched patients and logistic regression analyses of the secondary outcomes in the PEG and TPN groups are shown in **Table 3**.

**Table 3:** Secondary outcomes of propensity-matched patients (55 pairs) in the PEG and TPN groups.

Outcome	PEG	TPN	^a^ *P*-value	^b^ Risk difference	^c^ Odds Ratio	^d^ *P*-value
	*n* (%)	*n* (%)		% (95% CI)	(95% CI)	
Oral intake recovery	4 (7.3)	3 (5.5)	1.00	1.8 (−7.3, +10.9)	1.36 (0.29–6.38)	0.70
Discharge to home	7 (12.7)	4 (7.3)	0.53	5.5 (−5.7, +16.6)	1.86 (0.51–6.76)	0.35
Severe pneumonia	28 (50.9)	14 (25.5)	0.010	25.5 (+7.9, +43.0)	3.04 (1.36–6.79)	0.007
Sepsis	6 (10.9)	17 (30.9)	0.018	−20.0 (−34.7, −5.3)	0.27 (0.098–0.76)	0.013

^a^ *P*-value for Fisher’s exact test is shown.

^b^ The risk difference for the PEG group with reference to the TPN group is shown.

^c^ ORs for the PEG group with reference to the TPN group are shown.

^d^ *P*-value for logistic regression analyses is shown.

There were no significant differences in the rates of oral intake recovery and discharge to home between the groups. The incidence of severe pneumonia was significantly higher in the PEG group (50.9% vs 25.5%, *P* = 0.010), whereas the incidence of sepsis was significantly higher in the TPN group (10.9% vs 30.9%, *P* = 0.018).

ORs for the PEG group with reference to the TPN group for severe pneumonia and sepsis were 3.04 (95% CI: 1.36–6.79) and 0.27 (95% CI: 0.098–0.76), respectively.

**Fig 2:**
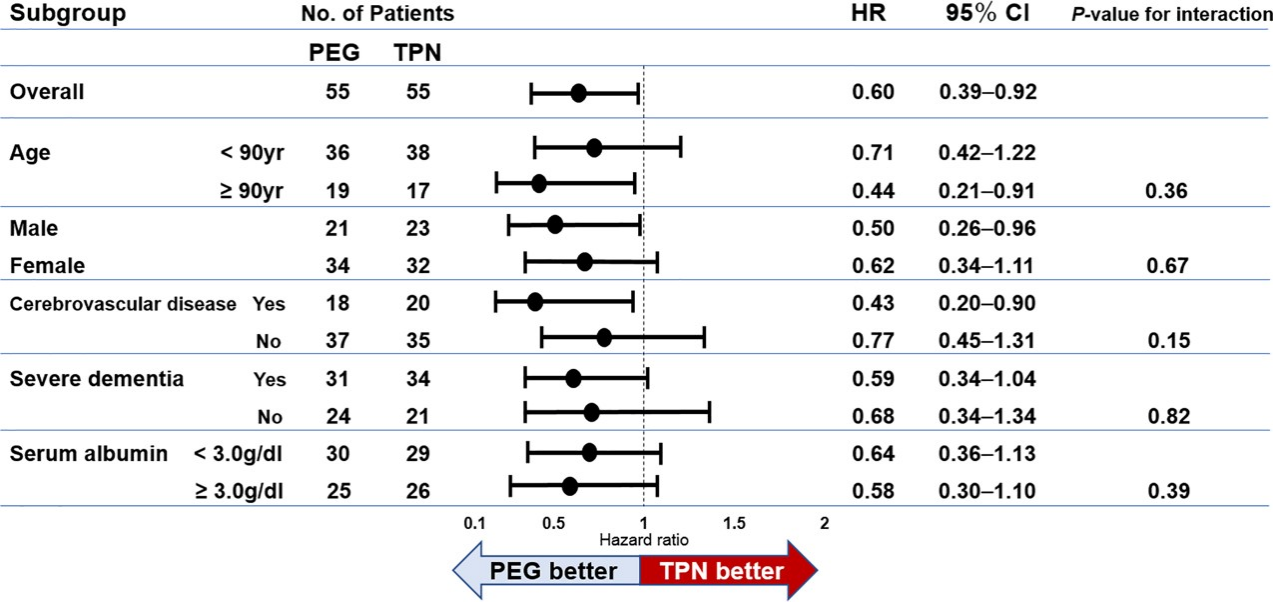
A forest plot of hazard ratios (HRs) for survival in the different subgroups. HRs from the subgroup analysis for survival between PEG and TPN are shown. HRs of < 1.00 indicate better survival in PEG compared with TPN.

Subgroup analysis for survival is shown using a forest plot in **Fig 2**. There is a trend that PEG showed better survival compared to TPN with statistical significance in the overall comparison, especially in patients aged 90 years and above, males and those with cerebrovascular disease.

The most frequent cause of death in the PEG and TPN groups was severe pneumonia (30.9%) and sepsis (16.4%), respectively.

## Discussion

This study investigated the long-term outcomes after PEG feeding and TPN in older patients using propensity score-matched analysis. We found that patients with greater age, lower nutritional state, and severe dementia were more likely to receive TPN, whereas patients with cerebrovascular disease were more likely to receive PEG. Survival time was significantly longer in the PEG group. The incidence of severe pneumonia was significantly higher in the PEG group whereas sepsis was significantly higher in the TPN group.

Previous studies that compared the outcomes of older patients with dysphagia managed with enteral nutrition and parenteral nutrition demonstrated consistent results stating that enteral nutrition was associated with lower mortality rates [10,30,31]. It has also been demonstrated that enteral nutrition is associated with a lower risk of systemic bacterial infection [30] and a higher rate of pneumonia compared with parenteral nutrition [31]. The general rule is that enteral feeding should be considered in patients with normal digestive function, whereas TPN should be used if enteral nutrition is not feasible [32,33]. Contrastingly, artificial nutrition for older patients with dysphagia can be a life-prolonging treatment [34–36]; therefore, especially in Japan, the choice of enteral versus parenteral nutrition is not only based on the digestive function of the patients but also on their clinical condition and the preferences of the patients and their family members [10,15,30,31]. This may result in selection bias and differences in the baseline characteristics of the PEG feeding and TPN study groups; therefore, we performed propensity score matching to adjust baseline characteristics to compare the effect of PEG feeding and TPN more accurately [16,26–28,31].

In this study, a comparison of baseline characteristics between the groups before propensity score matching revealed that patients with greater age, lower serum albumin levels, higher C-reactive protein levels, and severe dementia were more likely to receive TPN. Greater age, lower serum albumin levels, higher C-reactive protein levels, and severe dementia were reported as poor prognostic factors after PEG [5,6,20–22]. Our results indicated that PEG tended to be avoided in patients with such poor prognostic factors, and as a result, TPN was chosen as the alternative modality for artificial nutrition. Furthermore, TLC, TC, and hemoglobin were significantly lower in the TPN group than in the PEG group before propensity score matching, suggesting that TPN tended to be chosen for patients with a poorer general condition. Thus, in Japan, TPN is used as an alternative to PEG for older persons with dysphagia under the misconception that only PEG is an unnecessary life-prolonging treatment while TPN is not [15]. This practice lacks clinical evidence, and furthermore, the guidelines do not suggest TPN in place of PEG [33–36].

Survival analysis showed better results in the PEG group than in the TPN group. This may be explained by the fact that enteral nutrition has gastrointestinal, immune, and metabolic benefits compared with parenteral nutrition [32,33]. Additionally, in this study, the daily calorie intake was higher in the PEG group than in the TPN group. This difference between the groups may have affected the results of the survival analysis. In the past, studies showed that PEG did not improve survival in patients with dementia [5,6], whereas current studies shows that dementia patients with PEG feeding can survive longer than previously thought [7,10,37]. Our results were consistent with the results of these current studies. In our subgroup analysis, TPN was not consistently superior to PEG in all subgroups. The appropriateness of an alternative use of TPN to PEG was not supported by our analysis.

Most of the previous studies that compared enteral and parenteral nutrition in patients with dysphagia defined survival and infection rates as the primary and secondary outcomes, respectively [30–32]. Here, we placed importance on quality of life after the start of artificial nutrition, and thus we chose oral intake recovery and discharge to home as the secondary outcomes. Previous studies showed that age and BMI were predictive factors of oral intake recovery in stroke patients with tube-feeding [38,39]. In this study, age and BMI were similar between the groups, and there were no significant differences in oral intake recovery between groups. Oral intake recovery rates were low in both groups, with most patients requiring continuous artificial nutrition. Moreover, there were no significant differences in discharge to home between groups, indicating that both PEG feeding and TPN were feasible in a home environment [9,33,37]. However, the proportion of patients being discharged to their homes was also not high in either group, suggesting that most of the older patients with dysphagia requiring artificial nutrition were bound to stay in long-term care facilities rather than their own homes regardless of receiving PEG feeding or TPN.

The incidence of severe pneumonia was significantly higher in the PEG group. This result was expected and clinically plausible because enteral nutrition administered via PEG poses a risk of gastroesophageal reflux and consequent aspiration pneumonia owing to the underlying pharyngeal and laryngeal dysfunction of patients who require feeding through this modality [31–33]. In contrast, as expected, the incidence of sepsis was significantly higher in the TPN group. This may be due to the fact that TPN has been associated with catheter-related bloodstream infections and bacterial translocation [32,33,40–42]. Furthermore, the use of NT-CVC for long-term TPN may affect the rate of catheter-related bloodstream infections and the incidence of sepsis in the TPN group [43,44].

Several limitations of this study should be acknowledged. First, this was a retrospective observational study without randomization; therefore, assignment to each group may have been biased. Although propensity score matching was used to adjust the differences in baseline characteristics, the results may still have been biased because of unmeasured confounders. Second, the results of this study are applicable only to these patients who were included in the paired analysis, and therefore the results may not be generalizable to a broader population. Third, certain patients in the PEG group received not only PEG feeding but also TPN depending on their clinical condition, and furthermore, information on whether artificial nutrition was given until the end of life or was withdrawn or switched to subcutaneous hydration near the end of life was not investigated. Fourth, the daily calorie intake was not equal between the groups and the nutritional component was not investigated. Fifth, this was a single-center study with a small sample size.

## Conclusions

In summary, we performed a propensity-matched analysis to compare the outcomes of PEG and TPN in older persons. We found that compared to TPN, PEG was associated with better survival and a higher incidence of severe pneumonia as well as a lower incidence of sepsis, with no significant inter-group differences noted in oral intake recovery and discharge to home. Further studies are required to determine the type of patients who will benefit most from artificial nutrition, and studies on educational strategies are required to overcome the misinformed practice of TPN use instead of PEG in older patients with dysphagia. Further studies should also be conducted to determine strategies to lower the risk of severe pneumonia and to determine which type of central venous access for TPN has the least risk of sepsis.
